# Electrical receptive fields of retinal ganglion cells: Influence of presynaptic neurons

**DOI:** 10.1371/journal.pcbi.1005997

**Published:** 2018-02-12

**Authors:** Matias I. Maturana, Nicholas V. Apollo, David J. Garrett, Tatiana Kameneva, Shaun L. Cloherty, David B. Grayden, Anthony N. Burkitt, Michael R. Ibbotson, Hamish Meffin

**Affiliations:** 1 National Vision Research Institute, Australian College of Optometry, Carlton, Victoria, Australia; 2 Department of Medicine, St Vincent’s Hospital Melbourne, The University of Melbourne, VIC, Australia; 3 School of Physics, The University of Melbourne, Parkville, VIC, Australia; 4 NeuroEngineering Laboratory, Department of Biomedical Engineering, The University of Melbourne, Australia; 5 Swinburne University of Technology, Faculty of Science, Engineering and Technology, Hawthorn, Victoria, Australia; 6 Department of Physiology, Monash University, Clayton, VIC, Australia; 7 Centre for Neural Engineering, The University of Melbourne, Australia; 8 Department of Optometry and Vision Sciences, Faculty of Medicine, Dentistry and Health Sciences, The University of Melbourne, Australia; University of Washington, UNITED STATES

## Abstract

Implantable retinal stimulators activate surviving neurons to restore a sense of vision in people who have lost their photoreceptors through degenerative diseases. Complex spatial and temporal interactions occur in the retina during multi-electrode stimulation. Due to these complexities, most existing implants activate only a few electrodes at a time, limiting the repertoire of available stimulation patterns. Measuring the spatiotemporal interactions between electrodes and retinal cells, and incorporating them into a model may lead to improved stimulation algorithms that exploit the interactions. Here, we present a computational model that accurately predicts both the spatial and temporal nonlinear interactions of multi-electrode stimulation of rat retinal ganglion cells (RGCs). The model was verified using *in vitro* recordings of ON, OFF, and ON-OFF RGCs in response to subretinal multi-electrode stimulation with biphasic pulses at three stimulation frequencies (10, 20, 30 Hz). The model gives an estimate of each cell’s spatiotemporal electrical receptive fields (ERFs); i.e., the pattern of stimulation leading to excitation or suppression in the neuron. All cells had excitatory ERFs and many also had suppressive sub-regions of their ERFs. We show that the nonlinearities in observed responses arise largely from activation of presynaptic interneurons. When synaptic transmission was blocked, the number of sub-regions of the ERF was reduced, usually to a single excitatory ERF. This suggests that direct cell activation can be modeled accurately by a one-dimensional model with linear interactions between electrodes, whereas indirect stimulation due to summated presynaptic responses is nonlinear.

## Author summary

Implantable neural stimulation devices are being widely used and clinically tested for the restoration of lost function (e.g. cochlear implants) and the treatment of neurological disorders. *Smart* devices that can combine sensing and stimulation will dramatically improve future patient outcomes. To this end, mathematical models that can accurately predict neural responses to electrical stimulation will be critical for the development of smart stimulation devices. Here, we demonstrate a model that predicts neural responses to simultaneous stimulation across multiple electrodes in the retina. We show that the activation of presynaptic neurons leads to nonlinearities in the responses of postsynaptic retinal ganglion cells. The model is accurate and is applicable to a wide range of neural stimulation devices.

## Introduction

Implantable neural stimulation devices have demonstrated clinical efficacy, from the facilitation of hearing for deaf people using cochlear implants [1] to the treatment of neurological disorders such as epilepsy, Parkinson's disease, and depression using deep brain stimulation [2]. Additionally, neural stimulators are being used clinically for the restoration of sight [3–5]. Most stimulating neuroprostheses operate in an open-loop fashion; they do not adjust the stimulation by sensing how the stimulation affects the system. Devices that can both sense and stimulate will enable the development of new implants that may offer tighter control of neural activation and lead to improved patient outcomes [6].

The success of future retinal prostheses may benefit greatly from the ability to control spatiotemporal interactions between stimulating electrodes. For example, this may allow the design of stimulation strategies that better approximate the spiking patterns of normal vision. To this end, mathematical models that can predict responses to electrical stimuli are critical. A successful approach for extracting visual receptive fields uses models estimated from optical white noise stimulation patterns, which predict retinal responses [7–9] and responses in visual cortex [10, 11]. These models use high-dimensional random stimuli and rely on the identification of a low-dimensional stimulus subspace to which the neurons are sensitive. The features, or receptive fields, describe the spatial, temporal, or chromatic (for light stimuli) components of the stimuli to which the neurons are most sensitive. The low-dimensional subspace is commonly identified using spike-triggered average (STA) and spike-triggered covariance (STC) analyses [7, 12, 13] but other methods, such as spike information maximization, can be used [14–17]. In all of the aforementioned models, a stimulus is projected onto a feature subspace and then transformed nonlinearly to estimate the neuron’s firing rate. Generally, the accuracy of the model depends on the accurate identification of the low-order subspace.

Our previous work [12] demonstrated that short-latency RGC responses to electrical stimulation could be accurately described using a single linear ERF, and similarly for cortical responses [18]. In Maturana et al. [12], short-latency intracellular recordings were analyzed (i.e., responses within 5 ms of stimulus onset for which synaptically mediated network effects were not apparent). In the present study, we used extracellular recording because this is currently the only clinically viable method to measure retinal signals. Due to the presence of stimulation artefacts, we analyzed long-latency activity (>5 ms from stimulation onset), which arises largely from the activation of retinal interneurons [19]. For such indirect activation, we find that ERFs often have multiple sub-filters that can be estimated using a Generalized Quadratic Model (GQM) [16], with maximum likelihood methods, to accurately identify the low-dimensional subspace. Such maximum likelihood approaches have been shown to outperform regular STC analysis, revealing additional feature dimensions and more accurately predicting responses [15–17]. We present an approach using the GQM to recover spatiotemporal ERFs during electrical stimulation of the retina.

This study explores RGC responses to biphasic charge-balanced pulse stimulation applied at three stimulation frequencies: 10, 20 and 30 Hz. Our work used large diameter stimulating electrodes because of their relevance to clinical application [5]. Moreover, we explore suprathreshold electrical pulses of a similar duration to those used clinically (0.5 ms per phase). Nonlinear responses in RGCs during light stimulation have been shown to arise from activation of presynaptic neurons [20–26]. Therefore, it is conceivable that electrical stimulation of the retina may also lead to nonlinear responses in RGCs. Indeed, our results demonstrate that electrical stimulation of the retina leads to a range of nonlinear excitatory and suppressive effects. The GQM accurately predicts nonlinear neural responses and outperforms models based on a linear subspace. When synaptic transmission is blocked, we show that the model becomes linear and can be described by a simpler one-dimensional description, as in our previous study [12].

## Results

### Light response and electrical stimulation

The responses from 77 RGCs were recorded. These included 16 ON, 35 OFF, and 12 ON-OFF cells; the remaining 14 recordings had no identifiable light response or the cell morphology could not be recovered (for intracellular recordings). However, the responses to electrical stimulation from the non-classified cells were analyzed to verify the ability of the model to deal with responses of arbitrary types.

**Fig 1:**
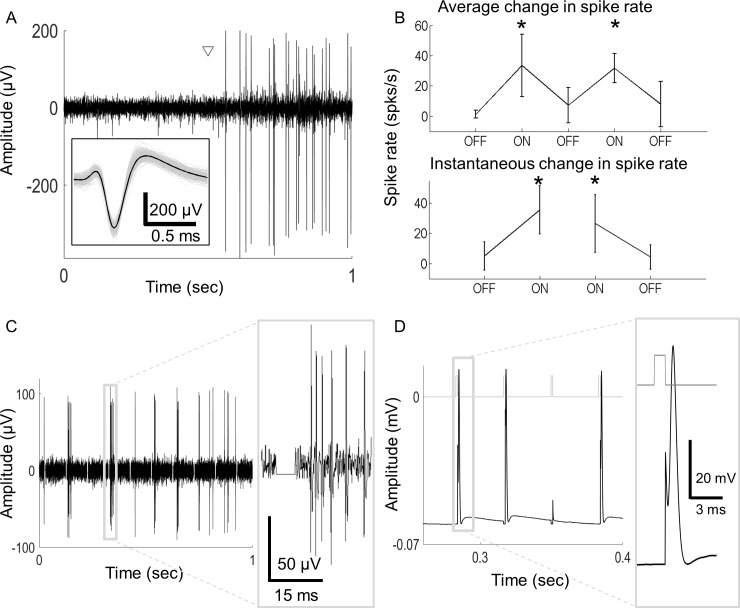
Sample light and electrical responses. A) An example ON cell responds with increased activity when light is switched on (onset marked with a triangle). The inset shows the overlaid spikes from a trial (gray) and the averaged response (black). B) The ON cell from A was stimulated with a spot of light centred at the electrode location. Light was switched on then off five times, each time for 10 s. The process was repeated seven times for this cell. Shown is the average spike rate during *light off* and *light on* periods (top) and the instantaneous spike rate 1 s prior to and after a change in illumination (bottom). Error bars represent standard deviations; * denotes a significant change in *light on* periods compared to *light off* (one-way ANOVA, p < 0.05). C) A sample cell showing the spikes in response to 10 electrical pulses over a 1 s period. Stimulation artefacts were removed online by blanking. Inset shows an expanded view of the stimulus artefact after removal. The remaining waveform reveals spikes detectable within 5 ms from stimulus onset. D) Intracellular recording showing action potentials (black trace) in response to electrical stimulation. Timing of electrical pulses is shown by traces at the top (grey). The expanded view shows how spikes can be detected with very low latency after artefact blanking.

Initially, cells were stimulated with a spot of light centered at the recording electrode location. Responses to light stimuli lasting 50 s (consisting of repeated periods of 10 s *light on*, 10 s *light off*) were recorded and repeated. The average spike rate and instantaneous change in spike rate were analyzed. A spike cluster analysis of the light response recordings confirmed whether the recordings were from single isolated cells. Fig 1A depicts a typical ON cell response during a transition from *light off* to *light on*. The white triangle shows the time when the light was switched on. This cell responded with more spikes during periods of *light on* compared to *light off* and produced more spikes during a transition from *light off* to *light on*. In this recording, smaller action potentials from another cell are also visible, however, these were not used. Fig 1B shows the average response during periods of *light on* and *light off* (top) and the instantaneous firing rate one second before and after transitions to *light on* and *light off* (bottom).

While we could not be certain that extracellular recordings were from the cell soma, in general, the recorded spikes had a distinctive biphasic shape with a large positive deflection towards the end of the spike (Fig 1A, inset). Studies have suggested that the origin of these spikes are somatic or proximal to the soma rather than distal axonal spikes [27, 28]. Stimulation artefacts were removed online using a 5 ms sample-and-hold circuit. High quality spikes between the stimulation pulses were observed, enabling online spike detection (Fig 1C). Note that the filter used during electrical stimulation had a much narrower band-pass range than that used during light stimulation; hence, the spike shape changed considerably (see Methods). The filter did not influence the frequency or timing of the spikes, only the appearance of the spikes.

Stimulation artefacts during intracellular recordings were also blanked (i.e. removed from the analysis) in a similar manner to extracellular recordings; however, this was only for the duration of the stimulation pulse (~1 ms). Using this technique, high quality action potentials with very low latency were recorded (Fig 1D).

### Model estimation

The major aim of this study was to develop mathematical models that could accurately relate RGC responses to arbitrary patterns of electrical stimulation. Specifically, we are interested in the influence of pre-synaptic neurons, which are known to produce nonlinear effects on RGCs [25]. To model RGC responses, electrical Gaussian white noise stimulation was applied to the retina using a 20-electrode array. The array was positioned subretinally; i.e. on the photoreceptor side of the retina.

Every RGC response was related to the applied stimulus over a 300 ms window preceding the response. An initial estimate of the spatiotemporal linear filters that affected the neuron’s response to electrical stimulation were obtained using spike-triggered average (STA) and spike-triggered covariance (STC) analysis (Materials and Methods). A second estimate of the spatiotemporal filters was obtained from the log-likelihood maximization of a General Quadratic Model (GQM, Eq (4), Materials and Methods) initialized with the significant filters obtained from STA/STC. Electrical pulses were applied at three stimulation frequencies: 10, 20 and 30 Hz. From the total of 77 cells, 30 cells were stimulated at 10 Hz, 22 cells at 20 Hz, and 44 at 30 Hz. Note that a subset of cells (11 of the 77) were stimulated at all three frequencies.

**Fig 2:**
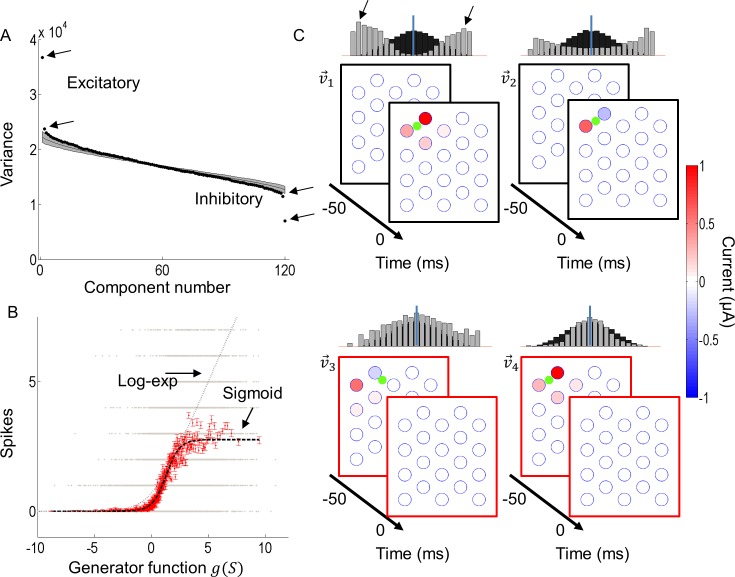
Model parameter estimation for a sample ON cell stimulated at 20 Hz. A) STC analysis revealed four significant components. Shown in black are all the eigenvalues, and the grey region denotes the null distribution of eigenvalues representing a 95% confidence interval. Eigenvalues that lie outside this region are considered significant (arrows). B) Stimuli along the generator function were plotted against the number of spikes that each stimulus produced (grey dots). These were binned along *g*(*S*) such that each bin contained 200 stimuli, from which the mean of each bin was computed (red dots with SE bars). A sigmoidal nonlinear function was fit to the binned data (black dashed curve). For comparison, a log-exponential fit is also shown (grey dashed curve). C) A maximum-likelihood estimation was carried out using the four components from A to initialize the estimation. The resulting excitatory components (${\vec{v}}_{1}$ and ${\vec{v}}_{2}$) and suppressive components (${\vec{v}}_{3}$ and ${\vec{v}}_{4}$) are shown. The green dots in the plots denote the approximate cell location. Above each plot is a histogram showing the input stimuli (black) and the ratio of responses to stimuli (grey) when projected onto each component.

The steps of the analysis for a sample cell stimulated at 20 Hz are shown in Fig 2. This ON cell produced four significant eigenvalues from the STC analysis, two of which were excitatory and two suppressive (Fig 2A). The four corresponding GQM component filters after log-likelihood maximization are shown in Fig 2C, where ${\vec{v}}_{1}$ and ${\vec{v}}_{2}$ represent the excitatory components and ${\vec{v}}_{3}$ and ${\vec{v}}_{4}$ represent the suppressive components. These filters can be interpreted as components of the electrical receptive fields (ERFs) of the cell. While a total period of *T* = 300 ms was explored, only stimulus frames in the period up to 50 ms significantly affected the neuron’s response. A linear component (${\vec{v}}_{0}$) was also computed but it was small, with no significant electrodes determined by a bootstrap test (see Materials and Methods) and hence not shown. The observed responses were binned and plotted against the generator signal, which integrates the output of the four filters via a quadratic nonlinearity (Eq (2) Materials and Methods; each bin contained 200 stimuli, red dots in Fig 2B). From this, a second nonlinearity could be estimated by fitting a sigmoid (Eq (5)) to the mean of each bin. A log-exponential fit is also shown for comparison, which shows a poor fit for high values of the generator signal.

Above each plot in Fig 2C are histograms showing the distribution of input stimuli (black) and the number of spikes per stimulus (grey) as a function of the stimulus projection onto each ERF component. For the excitatory components, the responses were bimodal, with ${\vec{v}}_{1}$ showing a slight preference towards negative amplitudes.

**Fig 3:**
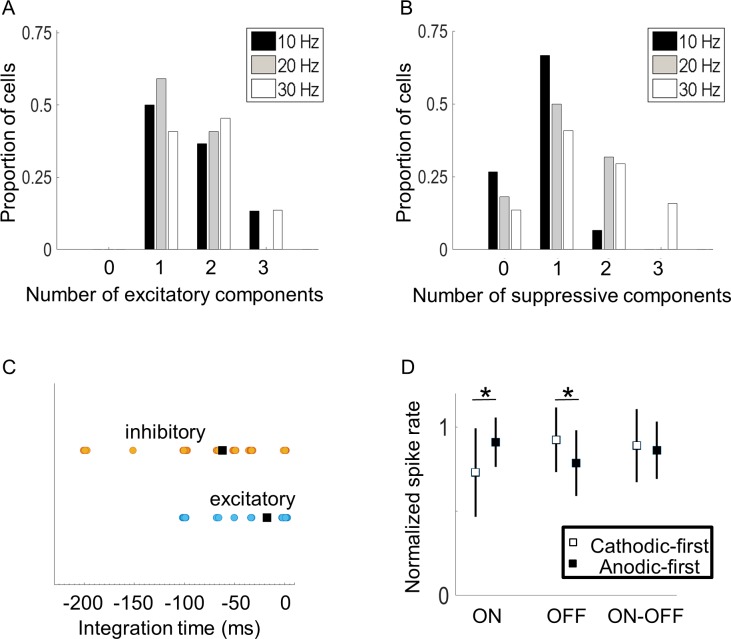
Electrical receptive field properties. A) Proportions of cells with up to three excitatory components. B) Proportions of cells with up to three suppressive components. C). The temporal windows over which suppressive and excitatory ERFs affected cell responses, thus indicating duration of stimulus integration time. Excitatory ERFs tended to occur within a short latency from the response (blue circles). Suppressive ERFs tended to extend over a long duration, which was variable from cell to cell (orange circles). The squares represent the means for all cells. D) RGC preference to cathodic-first or anodic-first stimulation. Squares represent means and lines indicate ±1 standard deviation. Stars denote significant differences (*p* < 0.05).

All cells had one or more significant excitatory components from STC analysis. Most also had significant suppressive components. Fig 3A and 3B summarizes the number of excitatory and suppressive ERF components for all cells. Stimulation at 20 Hz and 30 Hz tended to produce a larger number of significant components. The duration over which electrical stimulation affected a neuron’s response was unique to individual cells (Fig 3C). Electrodes that significantly contributed to a cell’s excitatory response tended to be constrained within a short latency from the response. For example, ${\vec{v}}_{1}$ and ${\vec{v}}_{2}$ in Fig 2B had significant electrodes only at a latency of 0 ms. Suppressive ERF components could extend up to 200 ms, but this varied across cells. For example, ${\vec{v}}_{3}$ and ${\vec{v}}_{4}$ in Fig 2B had significant electrodes at a latency of -50 ms. For the cell in Fig 2B, no components had significant electrodes that extended past -50 ms, hence this period constituted the integration time of this neuron. The average integration time for excitatory components was 22 (±40 SD) ms and for suppressive components was 58 (±54 SD) ms.

A major goal of electrical stimulation of the retina is to achieve selective stimulation of the ON and OFF retinal pathways. We compared responses between ON, OFF, and ON-OFF cells to see if any differences could be exploited. Excitatory ERFs, where all electrode amplitudes had the same polarity (i.e., ${\vec{v}}_{1}$ from Fig 2C), were compared to see if there was a cell-type specific preference to cathodic-first or anodic-first stimulation. To compare the preference to stimulus polarity, the normalized maximum spike-rate achieved for positive and negative amplitudes along the ERF was used (e.g. arrows in histogram from ${\vec{v}}_{1}$ from Fig 2C). Overall, a higher saturation rate was observed with anodic-first stimulation in ON cells (mean anodic-first 0.90 c.f. mean cathodic-first 0.73, *F*(1,27) = 5.02, *p* = 0.03, N = 14 ERFs), suggesting a higher dynamic range could be achieved with anodic-first pulses (Fig 3D). The opposite was observed in OFF cells; OFF cells preferred cathodic-first pulses (mean anodic-first 0.79 c.f. mean cathodic-first 0.92, *F*(1,77) = 10, *p* = 0.002, N = 39). No statistical difference was found among ON-OFF cells (*F*(1,27) = 0.14, *p* = 0.71, N = 12). No other differences (number of components, temporal or spatial characteristics) were found between ON, OFF or ON-OFF cells.

### Model validation

**Fig 4:**
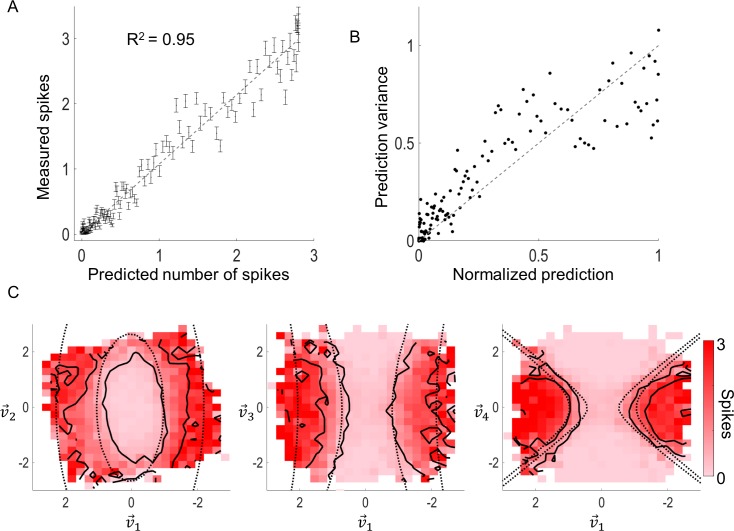
Model validation for a sample cell. A) The predicted response was compared to the average responses (black dots with SEM bars). Each point represents the mean response to 200 stimuli. B) The variance of each point in (A) was compared to the prediction. Both values were normalized to the maximum prediction (~3 spikes). For a Poisson-like process, the values should be equal (indicated by the dashed line). C) The contours of fixed response expectation (dotted black) were computed using the predicted model parameters (Eq (6)). These contours were compared to the contours generated from the experimental data (solid black) when projected onto the principal excitatory component and the second excitatory component (left), third excitatory component (middle), or a suppressive component (right). The contours denote the expectations of 1 and 2 spikes.

To test the accuracy of the model, we compared the model prediction (trained on 80% of data) against data from a validation set (20% of data) not used during estimation of the model parameters. Fig 4 shows the model validation for the example cell depicted in Fig 2. Fig 4A compares the prediction to the mean observed response; each point represents the mean response to 200 stimuli. The prediction and observed responses agreed very well (R^2^ = 0.95), producing an average error of approximately 0.12 spikes per bin (4.3% error relative to max response). By comparison, a best-case R^2^ value of 0.98 (computed by assuming a Poisson process, see Materials and Methods) was obtained.

To examine the assumption of Poisson spike train statistics, the variance of the responses was computed and normalized by the maximum predicted response at each bin location (Fig 4B). If a cell has Poisson spiking statistics, the variance should be equal to the mean (grey dashed line). For this example cell, the variance was approximately equal to the mean, which is consistent with Poisson-like statistics (R^2^ = 0.84, *F*(1,145) = 784, *p* = 3.9x10^-60^).

The contours of fixed response expectation (Eq (6), Materials and Methods) were computed using the predicted model parameters and compared to the measured response contours when the data was projected onto two of the linear filter components (Fig 4C). The experimental data conformed well to the curves predicted by the model, although this was noisy in the cases of the projections onto ${\vec{v}}_{2}$ and ${\vec{v}}_{3}$. This is most likely because these components were significantly smaller (see components in Fig 2A) than ${\vec{v}}_{1}$ and ${\vec{v}}_{4}$. This example also highlights a constraint of the GQM in that it assumes a symmetric quadratic nonlinearity, which may not be the case in general. However, the analysis provided a qualitative confirmation that this model is an appropriate description of neural responses to electrical stimulation. Moreover, it demonstrated that the data was highly nonlinear, and a model with a linear generator function is not an appropriate description of the spike probability.

**Fig 5:**
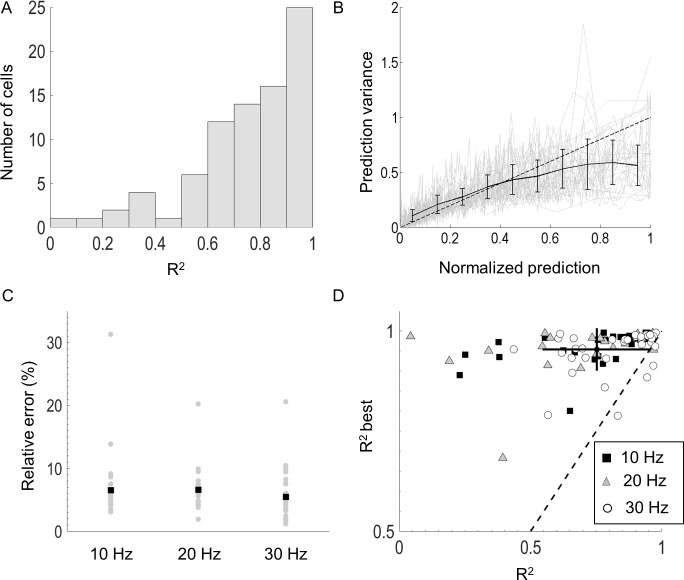
Model validation. A) The predicted response was compared to the observed responses from which a coefficient of determination was computed. Most cells achieved a high R^2^ value. B) The normalized prediction was compared to the normalized prediction variance (grey lines). A trend of lower prediction variance was observed for higher amplitudes of the generator function (black line). C) The perentage error relative to the maximal response for all cells (grey) at the three stimulation frequencies. Black squares denote the means. D) The measured R^2^ value was compared to the best case value computed by simulating a Poisson process with the estimated mean. The cross-hair denotes the average R^2^ for all cells (0.75) and the best case value assuming a Poisson process (0.94). The lengths of the lines denote ±1 standard deviation for each axis.

The model could accurately predict responses for most cells, demonstrated by a high coefficient of determination (Fig 5A). The average error for all cells was 0.14 spikes/bin (average of 6.45% error relative to max response). No significant differences were found between the model performance at 10, 20, and 30 Hz (Fig 5C). For each cell, the calculated coefficient of determination was compared to the best-case value, measured by simulating a Poisson process with a mean given by the model prediction. Overall, most cells achieved a high coefficient of determination (Fig 5D, mean R^2^ = 0.75). In all cells where R^2^ was below 0.5, relatively few spikes were collected (<10,000 spikes, c.f. average ~30,000 spikes for all other cells).

For many cells, the responses were Poisson-like; the variance of the response was approximately equal to the mean (Fig 5B, grey). However, a trend of lower variance at high amplitudes was observed (Fig 5B, black), suggesting that stimulating in a binary manner (zero or maximum current) rather than using intermediate amplitudes could lead to responses with lower relative variance.

### Origin of nonlinear responses

RGCs are known to spatially integrate light stimuli in a nonlinear fashion, which is mediated by presynaptic neurons [25]. Electrical stimulation of the retina activates retinal interneurons [19, 29], which are likely to produce nonlinear responses. For this reason, our model included nonlinear subunits. This is in contrast with previously published models [12, 30, 31], that found that electrodes interact in a largely linear fashion. An important difference between the present study and the investigations by Jepson et al. [30] and Maturana et al. [12] is that the latter only considered short latency spikes that occurred generally <5 ms from stimulus onset. In contrast, the extracellular recordings reported above were all long-latency spikes (>5 ms). If the origin of the nonlinear subunits is indeed a result of presynaptic neurons, then abolishing presynaptic input should recover a linear model similar to previous investigations if the short-latency spikes are recorded.

**Fig 6:**
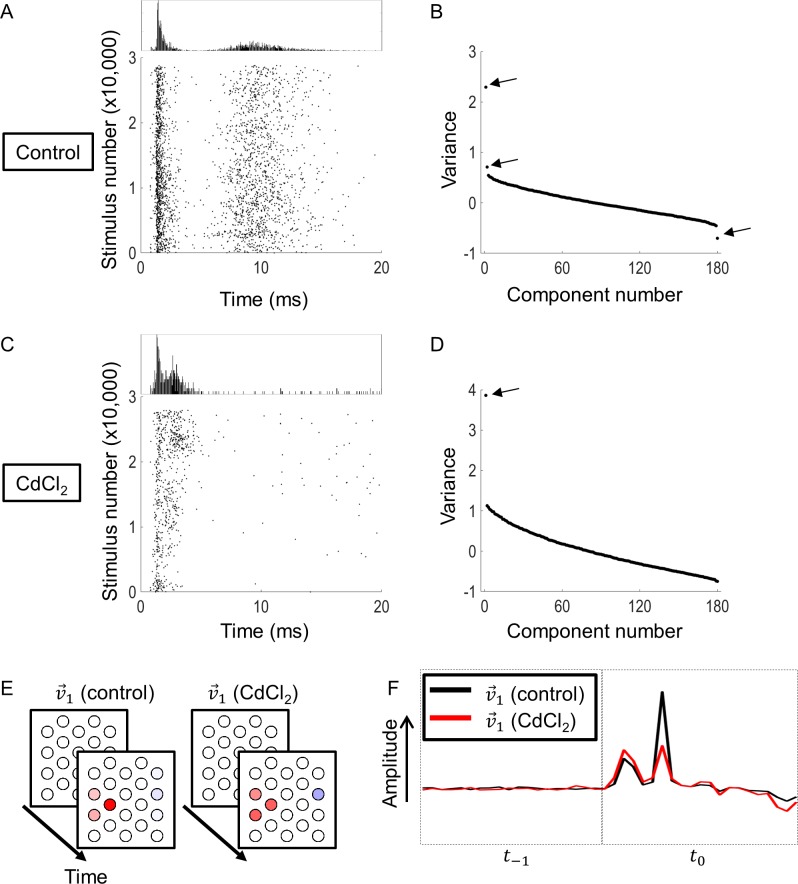
The effect of synaptic blockers. A) A raster plot of spike times for a sample cell prior to application of CdCl_2_. B) The eigenvalues produced from STC analysis showing three significant eigenvalues (arrows). C) A raster plot of spike times for the same cell as in (A) after application of CdCl_2_. While spontaneous spikes can be seen at times, long-latency activity was mostly abolished. D) The eigenvalues produced from STC analysis showing only one significant eigenvalue. E) The ERFs corresponding to ${\vec{v}}_{1}$ before (left) and after (right) application of CdCl_2_. F) A comparison of the electrode amplitudes making up the ERFs of ${\vec{v}}_{1}$, before and after application of CdCl_2_.

To test this, we recorded from nine cells using an intracellular patch clamp (which allowed the recovery of short-latency spikes), with normal Ames extracellular solution mixed with 250 μM CdCl_2_, which interferes with the release of neurotransmitters from nerve terminals. Fig 6A and Fig 6C compare raster plots of the spike times of a sample cell before and after the application of CdCl_2_. Prior to CdCl_2_, the histogram of spike times shows a distinct long latency peak, which is abolished by the addition of CdCl_2_, leaving only the short latency peak. This confirms that the long latency spikes are of presynaptic origin (>5 ms) while short latency spikes are the result of direct activation (<5 ms), and is consistent with previous studies [19, 32]. Fig 6B shows the eigenvalues from STC analysis of the responses, which shows three significant components. All but the first component disappeared after application of CdCl_2_ (Fig 6D), suggesting that two components, including the suppressive component, were of presynaptic origin.

Importantly, the reduction from two or more to a single component simplifies the model from a nonlinear to a linear generator function. To see this, we note that a model of the form

$$E(\vec{S_{t}})=N(\vec{v_{1}}.\vec{S_{t}},\vec{v_{2}}.\vec{S_{t}},\ldots ,\vec{v_{N}}.\vec{S_{t}})$$

is composed of a nonlinear function of a generator potential, which is in turn a function of the stimulus subspace:

$$E(\vec{S_{t}})=N\;\left(g(\vec{v_{1}}.\vec{S_{t}},\vec{v_{2}}.\vec{S_{t}},\ldots ,\vec{v_{N}}.\vec{S_{t}})\right).$$

The generator potential, $g(\vec{v_{1}}.\vec{S_{t}},\vec{v_{2}}.\vec{S_{t}},\ldots ,\vec{v_{N}}.\vec{S_{t}})$, is akin to the cell membrane potential, while the nonlinearity captures the conversion of this potential into an expected spike rate. In the simplest case, if the generator potential is linear in each argument, $\vec{v_{N}}.\vec{S_{t}}$, then it can be rewritten as a one-dimensional linear subspace: $g=\sum (w_{in}\vec{v_{N}}.\vec{S_{t}})=(\sum w_{n}\vec{v_{N}}).\vec{S_{t}}=v_{\mathrm{new}}.\vec{S_{t}}$, where $v_{\mathrm{new}}=\sum w_{n}\vec{v_{N}}$ defines the new one-dimensional subspace. If, however, the generator potential is a nonlinear function of two of more augments, then the subspace remains of two or more dimensions. In other words, a generator function of two or more dimensions is nonlinear except in the case where it can be written as a linear sum, thereby becoming one-dimensional.

**Fig 7:**
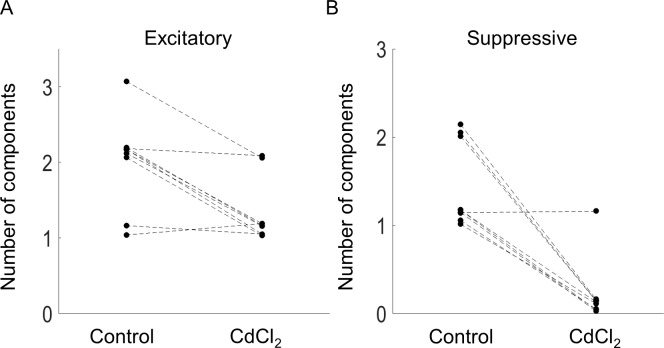
Number of excitatory (A) and suppressive (B) components before and after application of synaptic transmission blockers (CdCl_2_).

Application of CdCl_2_ reduced the number of significant components observed in the nine cells, simplifying the model. Fig 7 shows the numbers of significant components before and after application of CdCl_2_. Seven of nine cells were reduced to a single significant excitatory component, and eight of nine cells were reduced to zero suppressive components. This result is consistent with our previous observation [12], which showed that short-latency responses always produced a significant excitatory component, and sometimes some other small significant components. However, omission of these smaller components did not alter the accuracy of a model with a single linear filter. Application of CdCl_2_ also altered the duration over which the neuron integrated electrical stimulation. After application of CdCl_2_, the average integration time was reduced from an average of 89 ms to 44 ms.

### Comparison to other models

**Fig 8:**
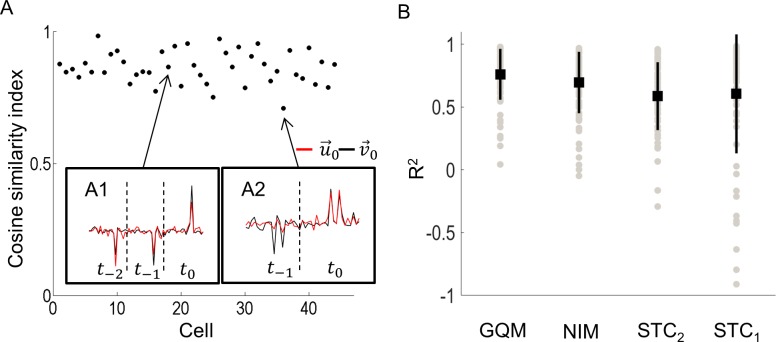
Cosine similarity index between ${\vec{v}}_{0}$ and ${\vec{u}}_{0}$. A) Similarity index for all cells. A1) Example cell with a high similarity index (0.88). ${\vec{v}}_{0}$ (black) was approximately equal to ${\vec{u}}_{0}=0.97{\vec{v}}_{1}-0.19{\vec{v}}_{2}-0.14{\vec{v}}_{3}$ (gray). A2) Example cell with the lowest similarity index (0.70). ${\vec{v}}_{0}$ (black) was approximately equal to ${\vec{u}}_{0}=0.71{\vec{v}}_{1}$ (gray). B) Comparison of four models. The coefficient of determination (R^2^) produced by the general quadratic model (GQM) was compared to the non-linear input model (NIM), a two-dimensional spike-triggered covariance analysis (STC_2_), and a linear model (STC_1_). Black squares denote the mean R^2^ and the lines represent 1 standard deviation. GQM performed significantly better than STC_2_ and STC_1_ (*p* < 0.03 in both cases).

To test if the GQM presented here was comparable or better than other models, we tested the performance of the GQM against other commonly used models. First, we compared the subspace spanned by the linear component ${\vec{v}}_{0}$, to the subspace spanned by the higher order components. It is possible that ${\vec{v}}_{0}$, which represents a linear approximation to the quadratic model, may span part of the stimulus space that is not included in higher order components. To test this, we compared the linear filter, ${\vec{v}}_{0}$, to higher order quadratic components from the GQM by estimating how much of ${\vec{v}}_{0}$ lies in the subspace spanned by the higher order components. Fig 8A show ${\vec{v}}_{0}$ for two sample cells along with the weighted sum of higher order components (${\vec{u}}_{0}$, Eq (7)). In both cases, ${\vec{u}}_{0}$ and ${\vec{v}}_{0}$ appear very similar, demonstrating that ${\vec{v}}_{0}$ spans the same subspace as the higher order components. For all cells, a high cosine similarity index was produced between ${\vec{u}}_{0}$ and ${\vec{v}}_{0}$.

Next, we compared the coefficient of determination between the predicted and measured responses for three other models, on a data set not used to fit the model. The GQM presented here was compared to a linear model using a projection onto the first STC component [STC1, 12], a two-dimensional STC analysis [STC2, 33], and the NIM [15] (Fig 8B). A linear model using only the STA is not shown, since for most cells it could not predict the responses. The main reason for this was that most cells were sensitive to both polarities of stimulation, leading to an almost zero STA.

Similar to the GQM model, both STC and NIM models incorporate a sum of non-linear receptive field terms, but do not make the assumption that the non-linearity is quadratic. While this makes them more general, it also means they can require more data to extract the same effective number of receptive fields. On average, the GQM produced a higher coefficient of determination than the other three models (Fig 8B). The GQM performed significantly better than both STC_1_ (*F*(1,191) = 10.03, *p* = 0.002) and the STC_2_ (*F*(1,204) = 5.03, *p* = 0.026, 1-way ANOVA). No significant differences were found between the GQM and NIM models.

### Responses to structured stimuli

**Fig 9:**
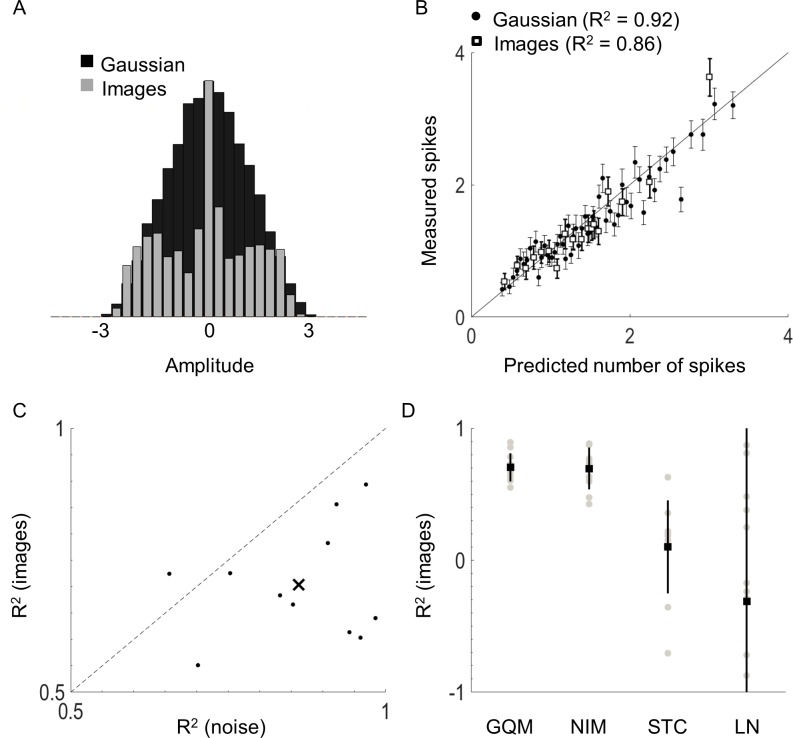
Model comparison with non-Gaussian stimuli. A) Distribution of electrode amplitudes for Gaussian and non-Gaussian image stimuli. The amplitudes in both cases have been divided by the variance of the Gaussian stimuli (i.e., the Gaussian distribution has unit variance). B) The model prediction for a sample cell is shown for Gaussian stimuli (round) and images (square). C) Coefficient of determination (R^2^) of the model prediction when validated with noise and with images for eleven cells. The x denotes the mean R^2^ for noise (0.86) and images (0.70). Dashed line represents line of equality. D) A comparison of the coefficients of determination for the general quadratic model (GQM), non-linear input model (NIM), a two-dimensional spike-triggered covariance analysis (STC_2_), and a one-dimension model (STC_1_). Squares denote the mean and lines denote ±1 standard deviation.

Patterns of electrical stimulation in blind patients is likely to contain high order correlations that are not present in Gaussian stimuli. Our model should therefore be robust to other stimuli that contain correlations. To gauge the capacity of the model to predict responses to non-white stimuli, eleven cells were also stimulated with a set of structured stimuli generated from a series of images (see Materials and Methods). The images included static wave gratings, spots and lines, and gratings moving in random directions. Importantly, these stimuli contain second and higher order correlations between electrodes. For each cell, the model parameters were estimated from white noise stimuli and used to predict responses to the stimuli from the images. Fig 9A shows the distribution of the electrode amplitudes for Gaussian stimuli (black) and the images (grey) for a sample cell, demonstrating very different stimulus distributions between the two sets of stimuli, even without considering correlations between electrodes. With both types of stimuli, the model could accurately predict responses (Fig 9B), suggesting that the model generalized well for non-white and correlated stimuli. The average error for Gaussian and patterned stimuli was 0.12 spikes/bin (~3.6% error relative to maximum response) in both cases.

The accuracy of model predictions for Gaussian stimuli tended to be higher than for the images (Fig 9C). The GQM and NIM performed significantly better than an STC_1_ (*p* < 5x10^-5^ in both cases, 1-way ANOVA). Performance for the GQM and NIM were similar (*F*(1,21) = 0.02, *p* = 0.88, Fig 9D).

### Spatiotemporal ERFs

**Fig 10:**
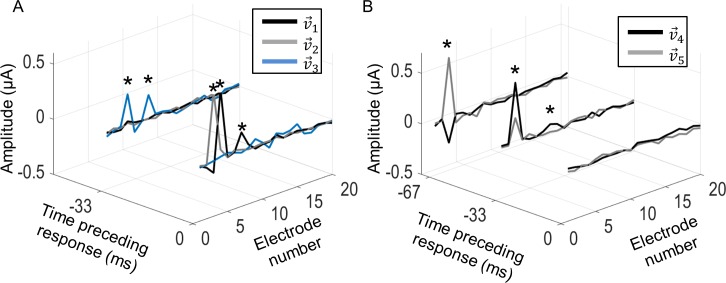
Spatiotemporal ERFs for a sample cell stimulated at 30 Hz. A) Three excitatory components were observed for this cell. Two components occurred in the same stimulus frame but were spatially different (${\vec{v}}_{1}$ and ${\vec{v}}_{2}$). The other occurred in a different stimulus frame (${\vec{v}}_{3}$). B) Two suppressive components were observed for this cell. The components were spatially similar to ${\vec{v}}_{1}$ and to each other but had different temporal characteristics. Although longer temporal windows were explored, only lags where significant electrodes occurred are shown. Stars denote electrodes that significantly affected the neuron’s response. Significance was calculated by a nested-bootstrap method (see Methods) at the 95% confidence level.

The model extracted diverse varieties of spatiotemporal ERFs. For most cells, the significant electrodes in the excitatory ERFs were limited to the stimulus frame immediately prior to the response. Cells that had more than one excitatory component tended to have electrodes that occurred in the same stimulus frame but were spatially different (e.g., ${\vec{v}}_{1}$ and ${\vec{v}}_{2}$, Fig 2B). However, some cells did have excitatory components with significant electrodes that extended over more than one stimulus frame. Fig 10A shows an example of a cell stimulated at 30 Hz that produced three excitatory components. The first two components (${\vec{v}}_{1}$ and ${\vec{v}}_{2}$) have significant electrodes in the same stimulus frame but are spatially different. The third component (${\vec{v}}_{3}$) is spatially aligned with ${\vec{v}}_{1}$ but has significant electrodes in a stimulus frame prior to that of ${\vec{v}}_{1}$.

Cells that produced significant suppressive components tended to be spatially aligned to the excitatory ERFs but occurred in preceding stimulus frames (e.g., ${\vec{v}}_{3}$ and ${\vec{v}}_{4}$, Fig 2B). However, suppressive components could also have significant electrodes in the same stimulus frames as the excitatory ones but with different spatial characteristics. Many cells produced suppressive components that extended over several stimulus frames. Fig 10B shows the suppressive components for the same cell in Fig 10A. Both ${\vec{v}}_{4}$ and ${\vec{v}}_{5}$ were spatially aligned, and aligned also to ${\vec{v}}_{1}$, but were not similar in their temporal characteristics to any excitatory component. Moreover, both suppressive components have significant electrodes at a lag of -33 ms, which is when the excitatory component, ${\vec{v}}_{3}$, occurred.

**Fig 11:**
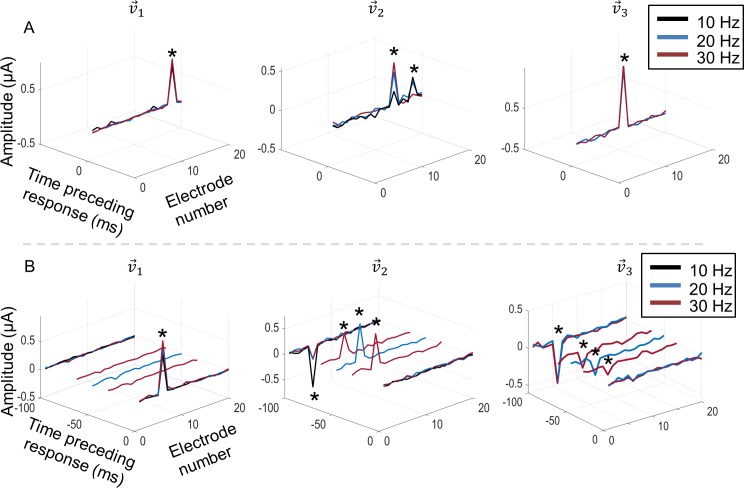
Effects of 10 Hz (black), 20 Hz (blue) and 30 Hz (grey) stimulation frequencies. A) Excitatory (${\vec{v}}_{1}$ and ${\vec{v}}_{2}$) and suppressive (${\vec{v}}_{3}$) components for a cell stimulated at three frequencies. Note that all components occurred at lag zero. B) Excitatory (${\vec{v}}_{1}$) and suppressive (${\vec{v}}_{2}$ and ${\vec{v}}_{3}$) components for another cell stimulated at three frequencies. In all plots, stars denote electrode amplitudes that significantly affected the neuron’s responses.

**Fig 12:**
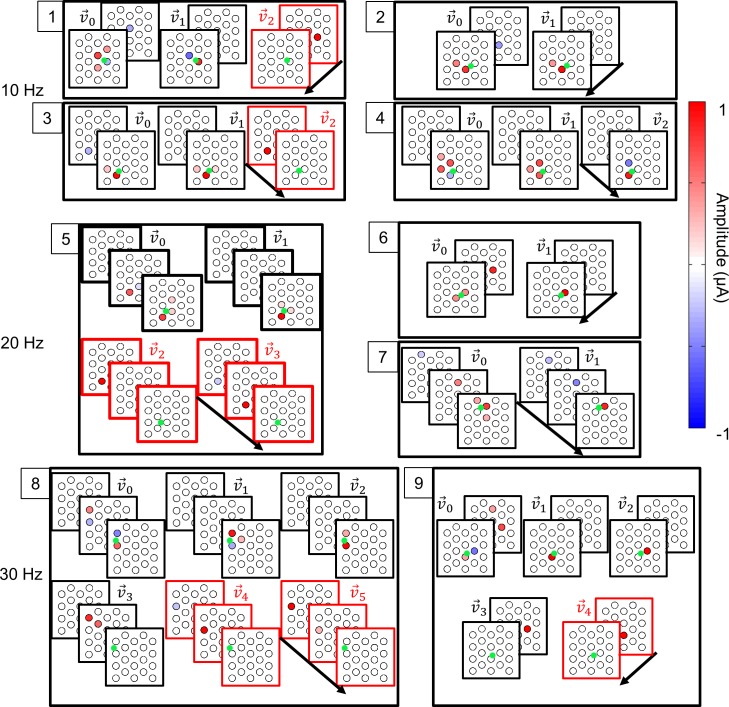
Sample of the diverse varieties of ERFs. Each numbered block represents the ERFs from a single cell. The ERFs in red represent suppressive components. The arrow represents the direction of time for each plot, with stimulus frames separated in time according to the stimulation frequency. Cells 1–4 were stimulated at 10 Hz, cells 5–7 at 20 Hz, and cells 8–9 at 30 Hz. Only significant electrode amplitudes are shown, and all significant electrodes are visible. Stimulating electrodes are separated by 1 mm center-to-center.

To test the effect of stimulation frequency, responses from a subset of 11 of the 77 cells were compared at the three stimulation frequencies: 10, 20 and 30 Hz. In all cases, the ERFs were similar across frequency, suggesting that stimulation frequency had little influence on the shape or temporal properties of the ERFs. Fig 11 demonstrates two example responses that are representative of all cells in the subset. The cell in Fig 11A produced two excitatory components (${\vec{v}}_{1}$ and ${\vec{v}}_{2}$) and a single suppressive component (${\vec{v}}_{3}$). All components occurred in the same stimulus frame (lag zero) but were spatially different. Across the three stimulating frequencies, the components were very similar, with the greatest difference occurring for ${\vec{v}}_{2}$ at 10 Hz. The cell in Fig 11B produced one excitatory component (${\vec{v}}_{1}$) and two suppressive components when stimulating at 20 and 30 Hz (${\vec{v}}_{2}$ and ${\vec{v}}_{3}$). Due to the different stimulation rates, pulses occurred at different times preceding the response between 0 and 100 ms but coincided at these two times. Both ${\vec{v}}_{2}$ and ${\vec{v}}_{3}$ show a continuity of the significant electrode over this time period across frequencies, suggesting that the effect of stimulation frequencies is to simply sample from a single consistent ERF. Note also that, while ${\vec{v}}_{2}$ and ${\vec{v}}_{3}$ were different at intermediate lags between 0 and -100 ms, the two components were the same at -100 ms. Hence only one suppressive component (${\vec{v}}_{2}$) was observed when stimulating at 10 Hz. Fig 12 shows a sample of the various ERFs observed at the three stimulation frequencies. The green dots in the frames denote the approximate recording electrode locations and red frames denote suppressive ERF components. The average ERF size (0.42 ± 0.46 SD μm), was calculated by computing a weighted average distance between significant electrodes and the recording location [12].

## Discussion

The research presented here is motivated by the goal to develop accurate neural response models of multi-electrode stimulation. Our work used large diameter stimulating electrodes, and short duration pulses due to their clinical relevance [5]. We demonstrated a method to fit a generalized quadratic model to RGC responses during electrical stimulation. The methods described were used successfully to recover model parameters in all tested ON, OFF, and ON-OFF RGC types at three stimulation frequencies: 10 Hz, 20 Hz and 30 Hz. Our study provides three main contributions:

1. We demonstrated that many RGCs have non-linear electrical receptive fields (ERFs) with two or more components that interact as a sum of quadratic terms.
2. We revealed that RGCs possess a range of excitatory and suppressive ERFs that are independent of stimulation frequency. However, higher stimulation rates provide better sampling of the ERFs. By stimulating using frequencies with periods smaller than the integration time of the neuron, more ERFs can be produced for some cells.
3. We demonstrated that the nonlinearity in the generator function results from activation of presynaptic neurons. Abolishing the presynaptic activity recovered a simpler model where the generator function could be described linearly by a one-dimensional description.

Summation of nonlinear presynaptic subunits has been shown to give rise to the nonlinear receptive fields of RGCs during visual stimulation [20–22, 24, 25]. For example, Hochstein and Shapley [20] demonstrated that Y cells in the cat have a linear center response and a surround response modulated by nonlinear subunits, whereas X cell responses were linear. More recently, Turner and Rieke [25] found that a greater proportion of OFF RGCs in primate exhibit nonlinear spatial integration than ON cells. In contrast, our study found no differences in the proportion of nonlinear subunits in ON and OFF; both ON and OFF cells were similar in their nonlinear response characteristics. For example, the ON cell in Fig 2 produced 2 excitatory and 2 suppressive subunits. The discrepancy could be influenced by the type of stimulus. Natural light stimuli affects the retina in a complementary fashion [26], whereas electrical stimulation possibly leads to simultaneous activation of the ON and OFF pathways, which in turn could produce unnatural nonlinear effects.

Most cells stimulated at 10 Hz only produced one or two components (usually one excitatory and one suppressive). All 10 Hz suppressive components were spatially very similar to the excitatory ones but with significant electrodes in a preceding stimulus frame to the excitatory component. Many more ERF components were observed when stimulating at 20 and 30 Hz due to the stimulation operating within the integration time of the neurons (~100 ms). This could give rise to ERFs lasting over multiple stimulus frames (i.e. Fig 11B). ERFs with similar durations have also been observed in rats [34] and mice [31]. However, both those studies only explored the spike-triggered average, which is likely to result from a linear combination of both excitatory and suppressive non-linear ERF components, as confirmed here.

Excitatory ERF components tended to have significant electrodes found close in time prior to the response peak. Suppressive ERF components occurred earlier with respect to the response peak (Fig 3D). For many cells with suppressive components, the components were spatially similar to the excitatory component but occurred one or two stimulus frames earlier (i.e. Fig 2B). This result suggests that repeated stimulation with the same electrodes may cause suppression in some cells, which has been noted previously [19, 35]. Importantly, rather than high stimulation frequencies leading to strong suppression in all RGCs, our work demonstrates that excitatory and suppressive ERF components are fixed and independent of stimulation frequency. Only some cells exhibited suppression and the duration of suppressive effects was cell dependent. Hadjinicolaou et al. [36] previously suggested that a cell’s dendritic field size could contribute to its ability to maintain excitatory responses; cells with small dendritic field sizes were shown to maintain significantly higher efficacy to repeated stimulation than cells with large dendritic fields. Similarly, modelling studies have shown that RGCs with smaller diameter dendrites respond with a higher spike frequency for a given stimulus amplitude [37].

Selective activation of visual pathways using electrical stimulation is a major goal of retinal implants. Given the diversity of RGC responses to electrical stimulation [38], it is probable that difference could be exploited to selectively activate target pathways. Sekirnjak et al. [39] demonstrated that individual activation of target parasol cells was possible using high-resolution multi-electrode arrays in primates. However, it is not known whether other non-parasol RGCs may have also been activated. Using the same type of array and animal model, Jepson et al. [40] demonstrated that selective activation of approximately 50% of midget cells was also possible. Stimulation strategies that vary the shape of the stimulus waveform over time could be used to improve selectivity. For example, selective activation of ON or OFF brisk transient cells in rabbit retina has been demonstrated using amplitude modulation of high frequency (~2000 Hz) biphasic pulse trains [41]. Our work also found small but significant differences between ON and OFF responses. OFF cells tended to respond more to cathodic-first biphasic pulse stimulation, while ON cells tended to prefer anodic-first pulses (Fig 3D).

A number of studies have demonstrated that modulation of pre-synaptic neurons is best achieved with long duration pulses [19, 32, 42, 43]. However, our results demonstrate that activation of the pre-synaptic network is achieved even at short pulse durations (0.5 ms), similar to that used clinically. It is possible that longer pulses would lead to even more pronounced nonlinearities appearing in the model. Given that suppression in RGCs during electrical stimulation arises from activation of retinal interneurons such as bipolar and amacrine cells [29], it is plausible that the dynamics of network-mediated excitation and suppression might be RGC-type specific. Our work also demonstrates that suppression largely arises from pre-synaptic neurons; only one cell (out of nine) retained a suppressive component after application of a synaptic blocker mixture (Fig 7). By increasing the spatial sampling of the ERF, differences in network connectivity, cell morphology, and cell size can be potentially exploited. This opens the possibility that selective stimulation using a cell’s specific ERFs might be useful for targeting stimulation to specific RGC types.

There are some key differences presented here to the results of Maturana et al. [12] that are worth revisiting. First, the extracellular responses analyzed here were at longer latencies (>5 ms versus <5 ms). As a result, modeled responses in the present study were largely a result of network activity. Methods that can reliably generate network mediated responses are desirable for improved spatial acuity, as studies have suggested that long-latency activity generate more spatially localized percepts compared to direct responses [34, 44]. Direct responses tend to generate elongated percepts due to simultaneous activation of passing axons [45]. Second, the data obtained here was recorded at ~34°C compared to room temperature by Maturana et al. [12]. Increased temperature has been shown to lead to a significant increase in long-latency activity (Maturana et al. [46]). Although a few cells presented by Maturana et al. [12] had significant suppressive components, these components were very small and had poor spatial structure. As a result, short-latency responses could be accurately modeled by a linear generator function. In contrast, analysis in the current work of long-latency responses revealed nonlinear excitatory and suppressive components. Third, the model presented here was based on extracellular recordings. Clinically, this is the only practical way to measure neural signals with a long-term implanted device, making this study more relevant to patient studies. One major limitation of the current method (c.f. Maturana et al. [12]) is that the model only accounts for average spike rate, and does not capture detailed spike timing dynamics on a time scale shorter than the inter-pulse width. For example, spike latencies for cells stimulated at 10 Hz were variable and could be as long as 70 ms. Spike timing has been shown to be very important for visual coding and spikes occur more precisely than those expected from a Poisson process [47, 48]. Hence models that could also account for precise spike timing are beneficial.

### Comparison to other models

This work compared the performance of a General Quadratic Model (GQM) to three other models: a linear model using a one-dimensional projection, a two-dimensional STC analysis, and the Nonlinear Input Model (NIM). The performances of GQM and NIM were very similar; both models could accurately predict responses and the correlation between prediction and response was similar in both cases. In general, the number of quadratic components in the GQM and components in the NIM were the same. However, the time taken to fit the NIM parameters was considerably longer than for GQM and increased with the number of components.

The simplest description of the spike-triggered subspace is found by analyzing the spike-triggered average. Sekhar et al. [31] analyzed the STA produced by electrical stimulation of one electrode. The amplitudes of currents produced from the STA were found to change over time, often changing polarity from negative to positive or *vice versa*. Similarly, our results showed that for many cells stimulated at 20 and 30 Hz, the linear component, ${\vec{v}}_{0}$, produced significant electrodes that spanned a number of stimulus frames and changed polarity over time (i.e. Fig 8A). In contrast, the majority of excitatory or suppressive ERFs had significant electrodes that were confined to one stimulus frame. For all cells with a significant linear component, ${\vec{v}}_{0}$ could be accurately described as a linear combination of the quadratic components from the GQM. Also, the STA could be accurately described as a linear combination of significant STC components, suggesting that the higher order components spanned the same subspace as the STA. However, the advantage of using a model with second-order components is that the components separately characterize excitation and suppression and it can provide more predictive power by capturing the important nonlinearities in the responses, which are neglected by the STA.

## Materials and methods

### Retinal whole mount preparation and data acquisition

Methods conformed to the policies of the National Health and Medical Research Council of Australia and were approved by the Animal Experimentation Ethics Committee of the University of Melbourne (Approval Number: 1413306). Data were acquired from retinae of Long-Evans rats ranging from 1 to 6 months of age. The animals were initially anesthetized with a mixture of ketamine and xylazine prior to eye enucleation. After enucleation, the rats were sacrificed with an overdose of sodium pentobarbital (350 mg intracardial). Dissections were carried out in dim light conditions to avoid bleaching the photoreceptors. After hemisecting the eyes behind the ora serrata, the vitreous body was removed and each retina was cut into two pieces. The retinae were left in a perfusion dish with carbogenated Ames medium (Sigma) at room temperature until used. Pieces of retina were mounted on a multi-electrode array (MEA) with ganglion cell layer up and were held in place with a perfusion chamber and stainless steel harp fitted with Lycra threads (Warner Instruments). Once mounted in the chamber, the retina was perfused (4–6 mL/min) with carbogenated Ames medium (Sigma-Aldrich, St. Louis, MO) at 32–35°C. Recordings were obtained using two extracellular electrodes as described below in more detail, or using whole cell intracellular recordings.

**Fig 13:**
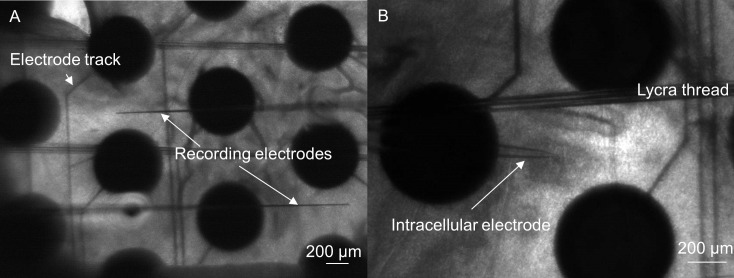
*In vitro* stimulation and recording. The retina was placed on a multi-electrode array (large black circles) and held in place with a perfusion chamber and lycra threads. A) Two extracellular electrodes were used to obtain recordings from the retinal surface. B) A hole was made in the inner limiting membrane to expose the RGCs. Once exposed, an intracellular glass electrode was used to obtain whole cell recordings of RGCs.

Extracellular potentials were recorded with custom-made carbon fiber electrodes (diameter ~7 μm) pulled in a glass pipette (Fig 13). The carbon fiber electrodes were used because they could reliably detect high-quality single cell spikes. Two electrodes were manipulated above the retinal surface (Sutter Instrument, MPC-200) until action potentials from both electrodes were obtained. Voltage recordings from two or more cells on each electrode were simultaneously collected, amplified, digitized with 18-bit precision at 50 kHz (Tucker Davis Technologies: RZ2 base station and PZ2 multichannel acquisition), and stored for offline analysis. Recordings lasting approximately 4 hours were obtained during each experiment.

Extracellular recordings were filtered and processed, and spikes were detected online using a custom-built circuit that was programmed into the digital signal processor on the Tucker Davis Technologies RZ2 base station. A first-order Butterworth band-pass filter with frequency range 1–5 kHz was used to make the stimulation artefact easier to remove, allowing for online detection of spikes. Stimulation artefacts were removed using a sample-and-hold circuit that captured the voltage prior to stimulation and held its value for 5 ms, such that a constant signal was recorded during stimulation. Spikes in the remaining waveform were detected and counted using threshold detection (see following sections for details).

For each extracellular recording, a signal-to-noise ratio (SNR) was calculated to assess the quality of the extracellular recording. The SNR was calculated by collecting all detected spikes and calculating the ratio of the spike amplitude to the standard deviation of the waveform noise [49],

$$SNR=\frac{\max\left(\bar{W}\right)-min(\bar{W})}{2\vartheta_{n}},$$ (1)

where $\bar{W}$ is the average time-course of the spike waveform and *ϑ*_*n*_ is the standard deviation of the noise. The SNR was calculated for every stimulus train. Recordings that produced SNR values lower than 4 were discarded.

To confirm the origin of the observed responses, intracellular recordings were conducted in some experiments. This allowed high-quality recording of low latency activity (<5 ms from stimulus onset), which is difficult to obtain with extracellular recordings. In these experiments, synaptic transmission was blocked after approximately 1 hour of recording responses in normal Ames medium. To block synaptic transmission, extracellular solution containing 250 μM Cadmium Chloride (CdCl_2_, Sigma-Aldrich) was applied. This agent was applied directly to the Ames medium and perfused over the retina. Intracellular action potentials were detected using threshold crossings after the stimulation artefacts were blanked (1 ms blanking). This allowed the detection of spikes with very low latency (<5 ms) as demonstrated previously [12].

Whole cell intracellular signals were recorded using standard procedures [50]. To obtain responses from RGCs, a small hole was made in the inner limiting membrane to expose some RGC somas. A pipette was then filled with internal solution containing (in mM) 115 K-gluconate, 5 KCl, 5 EGTA, 10 HEPES, 2 Na-ATP, 0.25 Na-GTP (mosM = 273, pH = 7.3), Alexa Hydrazide 488 (250 mM), and biocytin (0.5%). The initial pipette resistance in the bath was in the range 6–8 MΩ. Prior to recording, the pipette resistance was nulled using a bridge balancing circuit and the capacitance was compensated on the amplifier head stage. Voltage recordings were obtained in current-clamp mode, amplified (SEC-05X, NPI electronic), and digitized with 16-bit precision at 25 kHz (National Instruments BNC-2090).

### Light responses and cell identification

Cells were classified as ON, OFF, or ON-OFF by their responses to a spot of light (~100 μm diameter) centered at the recording electrode location. Cells were illuminated with stimuli lasting a total of 50 sec, consisting of alternating periods of light and dark, with each period lasting 10 sec. The stimuli were repeated 5–10 times. The average spike rate during dark periods was compared to the average spike rate during light periods. Additionally, the instantaneous change in spike rate from one second before to one second after a change in illumination was compared. Cells were classified as ON if they produced a larger number of spikes during *light on* periods compared to *light off* periods, or if they produced a greater number of spikes during the transition to a *light on* period. The opposite was true for OFF cells. Cells were classified as ON-OFF if they produced spikes during a transition from *light on* to *light off* and during a transition from *light off* to *light on*.

Extracellular action potentials in response to the light stimuli were recorded and filtered online using a first-order Butterworth band-pass filter with bandwidth 500–15,000 Hz. A threshold of 5 times the standard deviation of the recording was set for spike detection. Spikes crossing this threshold in the positive or negative direction were detected. To determine if detected spikes were from the same cell, we performed an offline spike cluster analysis (WAVECLUS, Quiroga et al. [51]) on the detected spikes from the light response recordings. WAVECLUS compares and clusters recorded spikes to determine which spikes are similar. Only recordings where single cells were identified were used.

### Electrical stimulation

Electrical stimulation was applied using an electrode array described previously [12]. The array was fabricated on a glass coverslip using lithographic methods to produce 20 platinum electrodes. Electrodes were spaced in a hexagonal arrangement with center-to-center spacing of 1 mm (Fig 13). Each electrode was circular with a diameter of 400 μm. The total stimulating area spanned approximately 3.5 mm x 3.5 mm.

Stimulation consisted of biphasic pulses applied simultaneously to all electrodes at a frequency of 10, 20 or 30 Hz. Pulses of equal phase duration (500 μs per phase) with an interphase gap (50 μs) and random amplitude were chosen independently for each electrode. The random amplitudes were sampled from a Gaussian distribution with standard deviation σ = 150 μA. A stimulus vector, $\vec{s}$, of length 20 (equal to the number of active electrodes) was generated by sampling each element from a Gaussian distribution. If the amplitude of stimulation of an electrode exceeded the stimulator limits (±300 μA), then the amplitude value was discarded and a new value was generated from the distribution. Positive stimulus amplitudes refer to anodic first stimuli, while negative amplitude refer to cathodic first. A stimulus train lasting 1 minute was generated and applied to the retina. The experiment continued for up to 4 hours with approximately 100,000 total stimuli applied at 10 Hz (proportionally more at higher frequencies). Stimulation waveform signals were generated by a custom-made MATLAB (MathWorks, version 2014a) interface commanding a multichannel stimulator (Tucker Davis Technologies: RZ2 base station and IZ2 multichannel stimulator).

### Mathematical model

Similar to Park and Pillow [16], we consider a General Quadratic Model (GQM) where a quadratic generator signal is used to map a stimulus onto the real line:

$$g(\vec{S_{t}})={\vec{v}}_{0}∙\vec{S_{t}}+\sum_{i=1}^{N}w_{i}{\left({\vec{v}}_{i}∙\vec{S_{t}}\right)}^{2},$$ (2)

where $\vec{S_{t}}$ represents the set of stimuli presented over the time period [*t*−*T*, *t*], ${\vec{v}}_{i}$ (*i* = 0,…,*N*) represent linear filters that span the relevant stimulus subspace, and *N* represents the dimension of the stimulus subspace. *w*_*i*_ is either +1 or –1 depending on whether ${\vec{v}}_{i}$ is excitatory (positive) or suppressive (negative). A 300 ms window was chosen for *T* since this has been shown to be a conservative estimate of the duration over which the retina integrates visual information and, hence, constitutes the “memory” of RGCs [7]. Furthermore, preliminary experiments showed that this captured the period over which stimulus history appeared to influence RGC responses to electrical stimulation. The spike rate was estimated by a nonlinearity operating on the generator signal:

$$E(\vec{S_{t}})=N(g(\vec{S_{t}})),$$ (3)

where $E(\vec{S_{t}})$ represents the expected number of spikes in response to stimulus $\vec{S_{t}}$.

The parameters of Eqs (2) and (3) were estimated by assuming that each neuron’s spikes were described by an inhomogeneous Poisson process with the firing rate function given by $E(\vec{S_{t}})$, and maximizing the log-likelihood of the model parameters given an observed set of responses:

$$L=\sum_{t}R(t)\;\log\;\left(E(\vec{S_{t}})\right)-E(\vec{S_{t}}),$$ (4)

where *R*(*t*) is the observed response at time *t*.

The optimization was carried out using the NIMtoolbox [15] in MATLAB. When the linear filters ${\vec{v}}_{i}$ (Eq (2)) were found, the components were orthogonalized and scaled according to their eigenvalues (such that the length of ${\vec{v}}_{i}$ was the square-root of its eigenvalue). The optimization procedure used in the NIMtoolbox assumes a log-exponential type nonlinearity for Eq (3). However, for our observed responses, the log-exponential nonlinearity was not an accurate fit for large values of the generator function, so a sigmoid nonlinearity was used instead:

$$N(g(x))=\frac{a}{1+\exp(-b(g(x)-c))},$$ (5)

where *a* represents a scaling factor that determines the saturation amplitude of the sigmoid, *b* represents the gain of the sigmoidal curve, and *c* represents the threshold (50% of the saturation level).

**Fig 14:**
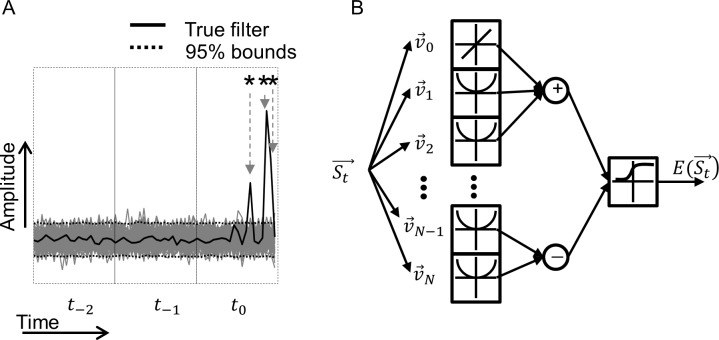
Significance testing of the electrode contributions to the electrical receptive fields (ERFs) and model summary. A) Three stimulus frames (*t*_−2_, *t*_−1_, and *t*_0_) of the true excitatory filter for a cell (black) are compared to a distribution of randomly time-shifted filters (grey). Each stimulus frame contains 20 amplitudes representing the amplitudes of the 20 stimulating electrodes. A 95% confidence limit is calculated from the distribution (dark grey) to which the actual values can be compared. In this example, three electrodes (denoted by stars) from *t*_0_ significantly affected the response of the neuron, and none from *t*_−1_ or *t*_−2_. B) A block diagram illustrating the steps of the GQM procedure. An input stimulus, $\vec{S_{t}}$, is linearly filtered and squared (for the case of ${\vec{v}}_{1}$ to ${\vec{v}}_{N}$). The outputs are then added or subtracted depending on whether the linear filter is excitatory or suppressive, respectively. An estimate of the response is produced by transforming the result with a sigmoidal nonlinearity.

To determine which electrodes in each receptive field significantly affected the cell’s response, the optimization procedure was repeated 1000 times using randomly time-shifted versions of the spike-triggered stimuli; i.e., randomly time shifting *R*(*t*) by *t*′. This gave a distribution for the linear and second-order components to which the true components could be compared. Electrode amplitudes that were greater in absolute value than two standard deviations were considered significant. Fig 14A depicts an example where a cell’s filter is compared to a distribution over three stimulus epochs (*t*_−2_, *t*_−1_, and *t*_0_). Each epoch represents the current amplitude applied to the 20 stimulating electrodes at the corresponding times preceding the response. A diagrammatic description of the whole model is shown in Fig 14B.

A fixed value of the generator function (Eq (2)) describes a quadratic surface of constant mean response. By projecting the stimulus-response data onto any two major components, the surface can be estimated in two dimensions by an ellipsoid (for excitatory-excitatory dimensions) or hyperbola (for excitatory-suppressive dimensions) with major and minor axes weighted by the strength (or norm) of each component. The equation describing this surface is:

$$g^{\prime }(x,y)=k_{z}+k_{x}+k_{y}+w_{i}x^{2}+{w_{j}y}^{2}$$ (6)

where $x={\vec{v}}_{i}∙\vec{S_{t}}$, $y={\vec{v}}_{j}∙\vec{S_{t}}$. *k*_*x*_ and *k*_*y*_ describe an offset from zero (due to a non-zero projection of ${\vec{v}}_{0}$ onto either ${\vec{v}}_{i}$ or ${\vec{v}}_{j}$ in Eq (2)), *k*_*z*_ describes a DC offset, and *w* are the same weights as in Eq (2). The contours of the surface described by *g*′(*x*,*y*) were used as a qualitative measure to test the suitability of the GQM by comparing it to the contours in the response data.

The simplest low-dimensional subspace that characterizes the neuron’s response can be described by the spike-triggered average (STA), ${\vec{v}}_{STA}$ [7]. This is found by taking the average stimulus that generated a response. ${\vec{v}}_{STA}$ can be used to capture the deviation between the spike-triggered and raw set of stimuli if the neuron has a nonlinearity such that the mean of the spike-triggered distribution is shifted away from the raw stimulus set. Second-order components that best capture the difference between the spike-triggered stimuli and the raw ensemble can be found by performing a spike-triggered covariance (STC) analysis [12, 13, 33]. Significant eigenvectors of the STC matrix span a stimulus subspace that generates an excitatory or suppressive influence on the neuron.

Prior to maximization of Eq (4), components of Eq (2) were initialized by ${\vec{v}}_{STA}$ for ${\vec{v}}_{0}$ and STC components for ${\vec{v}}_{i}$ (*i* > 0). The number of significant excitatory and suppressive components in Eq (2) (i.e., the dimensionality *N*) was found by incrementally increasing the number of excitatory and suppressive components until the best model performance was obtained. Model performance was measured by comparing the model prediction to the observed response from a validation set of data (see next section for details).

### Model validation

The accuracy of the model was evaluated by comparing model predictions to observed responses. The data from each cell was divided into five equal sets, four of which were combined to estimate the model parameters and one that was used to test the model’s prediction. To ensure that each result was not an effect of a particular choice of data, the results were cross-validated by repeating the analysis with a different set of the originally partitioned data. The mean values of the five cross-validated sets are reported. All reported *p* values were calculated using the Tukey-Kramer test unless otherwise stated.

**Fig 15:**
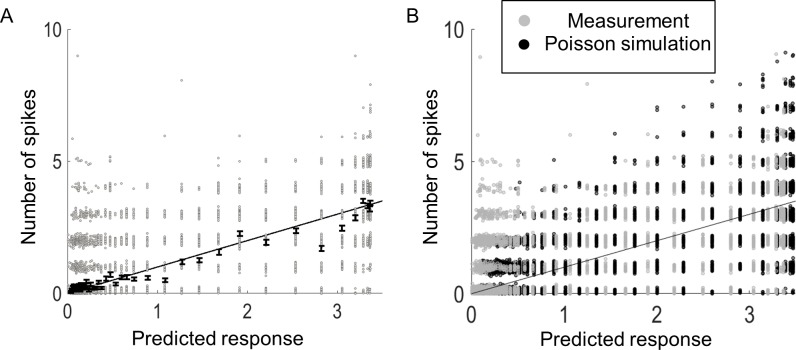
Example of the validation measures. A) Measured spikes from individual trials were binned (grey dots) and averaged (black dots with SE bars), and compared to the predicted response (black line). From the averaged responses, a coefficient of determination was computed (R^2^ = 0.93, *F*(1,54) = 1131, *p* = 2x10^-37^). B) The measured spikes (grey) were compared to a simulated Poisson process (black dots) with mean given by the predicted response (line). From the simulation, a coefficient of determination was calculated (R^2^ = 0.98, *F*(1,54) = 41x10^3^, *p* = 2x10^-78^). Note that the actual responses and simulated Poisson process are shown with jitter to illustrate the number of underlying responses.

Fig 15A demonstrates the validation for an example cell. The measured spike counts from the validation set (grey dots) are plotted against the predicted spike count (black line), binned into groups of 200 stimuli. The average spike rate in each bin (black dots with error bars) was used to compute an average prediction error. The average error was calculated as the mean difference between the predicted and measured spike count for each bin. For the example in Fig 15A, the average error is 0.15 spikes/bin (4.5% error relative to maximum response of 3.3 spikes). Additionally, a coefficient of determination between the mean response and the model fit was computed (R^2^ = 0.93 for the example in Fig 15A, *p* < 0.01). To obtain an upper bound of this value, a *best-case* coefficient of determination was estimated by assuming the neuron could be described by a Poisson distribution (this assumption is later tested). At each bin location, a Poisson process was simulated (black dots in Fig 15B), with a mean given by the model prediction (black line in Fig 15B). Qualitatively, both the raw data (grey dots) and Poisson simulated data were similar. From the simulated data, a coefficient of determination was computed and compared to the observed value. For the example in Fig 15B, the best-case coefficient of determination is 0.98.

The spike triggered average (STA) is often used to describe retinal responses to light [7] and to electrical stimulation [31, 52]. In these models, a linear kernel described by ${\vec{v}}_{STA}$ is used to model the neural responses. However, ${\vec{v}}_{STA}$ can often describe a linear combination of higher order components [17] and, therefore, may describe a combination of excitatory and suppressive stimuli. To test this, we compared the subspace spanned by the quadratic components in our model to the linear component, ${\vec{v}}_{0}$. We computed a weighted sum of higher order components, where the weights of each component were fitted to ${\vec{v}}_{0}$. The weighted vector was given by ${\vec{u}}_{0}$, such that

$${\vec{u}}_{0}+\vec{\epsilon }=V\vec{b}+\vec{\epsilon }={\vec{v}}_{0},$$ (7)

where $\vec{b}$ is a vector of weights that scale a linear combination of second order components (${\vec{v}}_{i}$) contained in the columns of the matrix *V*, and $\vec{\epsilon }$ is the orthogonal remainder. $\vec{b}$ can be found by the relation

$$\vec{b}={V^{T}\vec{v}}_{0},$$ (8)

given orthonormal components in *V*. To test for the similarity between ${\vec{v}}_{0}$ and ${\vec{u}}_{0}$, a cosine similarity measure was used, which is the cosine of the angle between two vectors. A similarity of 0° produces a value of 1; any non-zero angle produces a value less than 1. Only cells that produced a ${\vec{v}}_{0}$ with electrodes that contributed significantly to a cell’s ERF were used (approximately half the cells). Significance was determined by the nested bootstrap method described in the previous section.

Our model was compared to three other models. First, we compared our model to a linear model, described by projecting the spike-triggered data onto a single linear component. The linear component was found by taking the first excitatory component of an STC rather than the ${\vec{v}}_{STA}$ since, for many cells, the ${\vec{v}}_{STA}$ was close to zero. Furthermore, the first component from the STC produced a far more accurate prediction. The data was projected onto the component and a double sided sigmoid was fit to the projected data [12]. Second, we compared the GQM to an STC analysis using a two-dimensional nonlinearity. Only a two-dimensional model was used since including more components generally led to over-fitting and required more data for a robust prediction. The two-dimensional nonlinearity was estimated by projecting the spike-triggered data onto the two most significant components (whose eigenvalues departed most from the null distribution, usually the first and last). From this, a two-dimensional smoothed surface was fit in MATLAB. A *lowess* fit with span of 0.05 was chosen for the surface. Third, we compared the GQM to the nonlinear-input model [NIM, 15]. The NIM models the synaptic inputs from excitatory and suppressive subunits using half-wave rectifying-type nonlinearities. This model is a more generalized version of the GQM that makes fewer assumptions about the higher order nonlinearities, but results in more parameters that need fitting. Like the GQM, the optimal number of components in the NIM were chosen by incrementally increasing the number of excitatory and suppressive components until the highest coefficient of determination between prediction and measured response, in a cross-validated set, was recorded.

### Responses to structured stimuli

**Fig 16:**
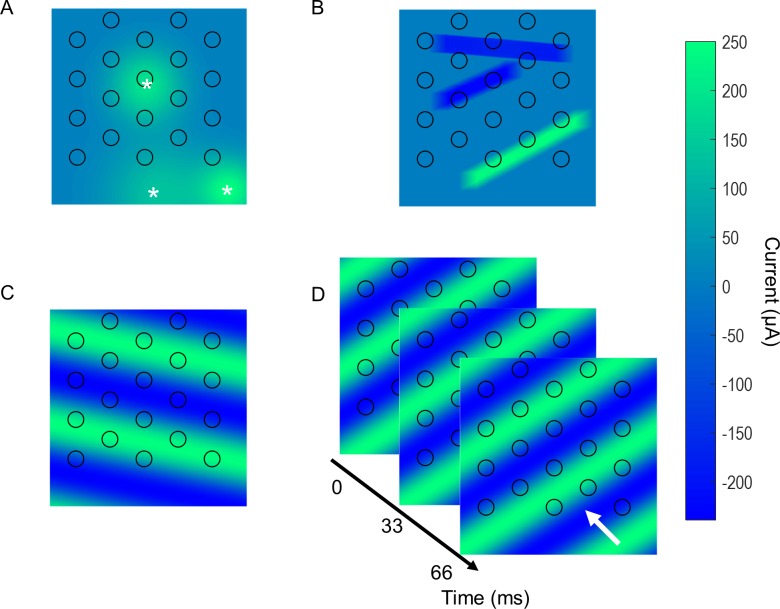
Examples of structured stimuli. A) Three spots placed in random locations. The white stars show the approximate center of each spot. B) Three lines placed in random locations and orientations. C) Stationary gratings with random spatial frequency and orientation. D) Moving gratings with random spatial frequency, orientation and temporal frequency. The white arrow shows the direction of movement.

To test the model’s capability to predict responses to structured, non-Gaussian stimuli, a subset of cells were activated with stimuli generated from a set of images. The images were randomly generated from one of the four following modes defined on a 500 x 500 grid. Each pixel in an image was assumed to span 10 μm, such that the entire 500 x 500 image spanned 5 x 5 mm. The following examples are shown in Fig 16:

1. Spots–Up to four spots appeared in the image in random locations. Spots were blurred with a Gaussian filter to give a smooth appearance. The maximum size of a spot prior to blurring was 1.5 mm. The upper spot in Fig 16A shows a spot of maximum size.
2. Lines–Up to four lines appeared in the image at random locations and orientations. Lines were blurred with a Gaussian filter to give a smooth appearance. Line lengths were randomly chosen, with the smallest length being 1 mm. The maximum limit was set to the image size.
3. Stationary wave grating–A sinusoidal wave grating image with random spatial frequency (below Nyquist limit set by inter-electrode separation) and random orientation (0–180°, steps of 1°).
4. Moving wave grating–A sinusoidal wave grating image with random spatial frequency (as in 3) moving in the direction of the wave with random temporal frequency (max 2 Hz). The moving grating lasted 300 ms.

All random features in modes 1–4 (i.e., spot locations, line orientations, etc.) were chosen using a uniform distribution. The orientations of lines and gratings were randomly chosen in the interval of 0–180° in steps of 1°. The speed of the moving wave grating was randomly chosen in steps of 0.1 Hz, up to a maximum of 2 Hz. Each image from modes 1–3 was scaled such that the highest amplitude pixel had a value of +250 μA or –250 μA. Each image stayed constant for 180 ms. For the moving wave gratings, the set of images were scaled such that the maximum pixel in the set of images was +250 μA or –250 μA. The stimulating electrodes were superimposed on each image in their correct locations and the pixel amplitude at the center of each electrode was applied to the corresponding electrode. Stimulation was applied at 30 Hz in all cases, with each stimulus train lasting 30 sec.
